# An Endogenous Murine Leukemia Viral Genome Contaminant in a Commercial RT-PCR Kit is Amplified Using Standard Primers for XMRV

**DOI:** 10.1186/1742-4690-7-110

**Published:** 2010-12-20

**Authors:** Eiji Sato, Rika A Furuta, Takayuki Miyazawa

**Affiliations:** 1 Laboratory of Signal Transduction, Institute for Virus Research, Kyoto University, 53 Shogoin-Kawaracho, Sakyo-ku, Kyoto 606-8507, Japan; 2 Japanese Red Cross Osaka Blood Center, 2-4-43 Morinomiya, Joto-ku, Osaka 536-8505, Japan

## Abstract

During pilot studies to investigate the presence of viral RNA of xenotropic murine leukemia virus (MLV)-related virus (XMRV) infection in sera from chronic fatigue syndrome (CFS) patients in Japan, a positive band was frequently detected at the expected product size in negative control samples when detecting a partial *gag *region of XMRV using a one-step RT-PCR kit. We suspected that the kit itself might have been contaminated with small traces of endogenous MLV genome or XMRV and attempted to evaluate the quality of the kit in two independent laboratories. We purchased four one-step RT-PCR kits from Invitrogen, TaKaRa, Promega and QIAGEN in Japan. To amplify the partial *gag *gene of XMRV or other MLV-related viruses, primer sets (419F and 1154R, and GAG-I-F and GAG-I-R) which have been widely used in XMRV studies were employed. The nucleotide sequences of the amplicons were determined and compared with deposited sequences of a polytropic endogenous MLV (PmERV), XMRV and endogenous MLV-related viruses derived from CFS patients. We found that the enzyme mixtures of the one-step RT-PCR kit from Invitrogen were contaminated with RNA derived from PmERV. The nucleotide sequence of a partial *gag *region of the contaminant amplified by RT-PCR was nearly identical (99.4% identity) to a PmERV on chromosome 7 and highly similar (96.9 to 97.6%) to recently identified MLV-like viruses derived from CFS patients. We also determined the nucleotide sequence of a partial *env *region of the contaminant and found that it was almost identical (99.6%) to the PmERV. In the investigation of XMRV infection in patients of CFS and prostate cancer, researchers should prudently evaluate the test kits for the presence of endogenous MLV as well as XMRV genomes prior to PCR and RT-PCR tests.

## Findings

Xenotropic murine leukemia virus (MLV)-related virus (XMRV), which resembles endogenous MLV, was discovered in prostate cancer patients in 2006 [1,2]. In 2009, a high incidence of XMRV infection was also documented in chronic fatigue syndrome (CFS) patients in the United States [3]. Since then, surveys on XMRV infection of CFS patients have been conducted in several countries [4-9]; however, there is a vigorous debate over conflicting results in CFS patients [10-12]. Moreover, recently, Lo et al. detected MLV-related viruses which are distinct from XMRV but resemble polytropic endogenous MLVs in CFS patients and healthy blood donors [13].

In studies investigating XMRV infection, a PCR approach to detect proviral DNA and/or a RT-PCR approach to detect viral RNA have been commonly employed [1,3-6,8,13-15]. We (the Japanese Red Cross [JRC]) have been studying the prevalence of XMRV infection in Japanese patients with prostate cancer and CFS as well as healthy blood donors. To study the presence of XMRV RNA in plasma from CFS patients, we selected a commercial one-step RT-PCR kit. In the pilot study, we encountered a puzzling result. A positive band was frequently detected at the expected product size in the negative control (water) using primer sets to detect a partial *gag *region of XMRV. We suspected that the test kit itself might have been contaminated with small traces of endogenous MLV genome or XMRV and attempted to evaluate the quality of the kit in two independent laboratories, in JRC and Institute for Virus Research (IVR), Kyoto University (Kyoto, Japan).

We used the following RT-PCR kits which were purchased in Japan: SuperScript^®^III One-Step RT-PCR System with the Platinum^® ^Taq High Fidelity Kit (Cat. no. 12574-030) (Invitrogen, Carlsbad, CA, USA) (abbreviated as Kit I); AccessQuick™RT-PCR Sysytem (Cat. no. A1701) (Promega, Madison, WI, USA) (abbreviated as Kit P); One Step RT-PCR Kit (Cat. no. PRO24A) (TaKaRa, Ohtsu, Shiga, Japan) (Abbreviated as Kit T); One Step RT-PCR Kit (Cat. no. 210210) (QIAGEN GmbH, Hilden, Germany) (Abbreviated as Kit Q).

**Figure 1:**
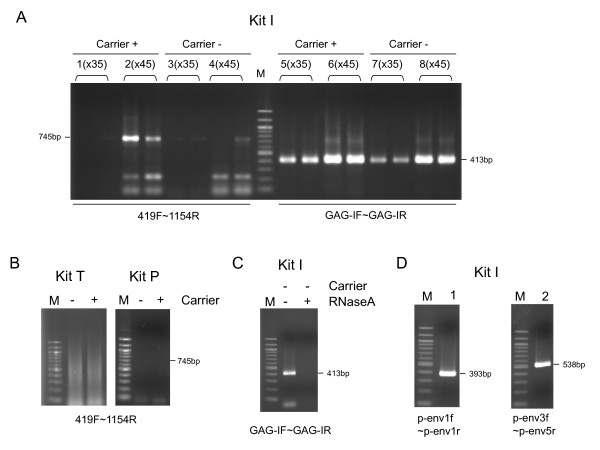
**Amplification of MLV-like viral sequences in Kit I**. (A) One-step RT-PCR was conducted using Kit I with the indicated primer sets. The RT-PCR conditions were as follows: reverse transcription at 55°C for 30 minutes; activation at 94°C for 2 minutes; 35 (lanes 1, 3, 5 and 7) or 45 cycles (lanes 2, 4, 6 and 8) of the following steps: 94°C for 15 s, 57°C for 30 s, and 68°C for 1 minute; and a final extension at 68°C for 3 minutes. Lanes 1, 2, 5 and 6: one-step RT-PCR with carrier RNA; Lanes 3, 4, 7 and 8: one-step RT-PCR without carrier RNA. Each reaction was carried out in duplicate. (B) One-step RT-PCR was conducted using Kit T (left panel) and Kit P (right panel) with primers 419F and 1154R with or without carrier RNA. The RT-PCR conditions using Kit T were as follows: reverse transcription at 50°C for 30 minutes; activation at 94°C for 2 minutes; 45 cycles of the following steps: 94°C for 30 s, 57°C for 30 s, and 72°C for 1 minute; and a final extension at 72°C for 10 minutes. The RT-PCR conditions using Kit P were as follows: reverse transcription at 45°C for 45 minutes; activation at 95°C for 2 minutes; 45 cycles of the following steps: 95°C for 30 s, 57°C for 30 s, and 70°C for 45 s; and a final extension at 70°C for 5 minutes. (C) One-step RT-PCR was conducted with primers GAG-I-F and GAG-I-R using Kit I with or without RNaseA. Carrier RNA was not added to the reaction mixtures. The RT-PCR conditions were as follows: reverse transcription at 55°C for 30 minutes; activation at 94°C for 2 minutes; 45 cycles of the following steps: 94°C for 15 s, 57°C for 30 s, and 68°C for 1 minute; and a final extension at 68°C for 3 minutes. (D) One-step RT-PCR was conducted using Kit I to amplify *env *region of the contaminants. One-step RT-PCR was carried out using two primer sets p-env1f and p-env1r (lane 1), and p-env3f and p-env5r (lane 2). The RT-PCR conditions were the same as in Figure 1C with the exception of the number of PCR cycles (60 cycles instead of 45 cycles). M: DNA size marker.

To amplify the partial *gag *gene of XMRV or other MLV-related viruses, primers 419F (5'-ATCAGTTAACCTACCCGAGTCGGAC-3') and 1154R (5'-GCCGCCTCTTCTTCATTGTTCTC-3') [3], and GAG-I-F (5'-TCTCGAGATCATGGGACAGA-3') and GAG-I-R (5'-AGAGGGTAAGGGCAGGGTAA-3') [1] were used. To amplify the partial *env *gene of polytropic endogenous MLV, primers p-env1f (5'-AGAAGGTCCAGCGTTCTCAA-3'), p-env1r (5'-TTGCCACAGTAGCCCTCTCT-3'), p-env3f (5'-GATGAGACTGGACTCGGGTG-3') and p-env5r (5'-GTGGAGGCCTGGGGAGCATGATC-3') were designed based on the sequence of a polytropic endogenous MLV (PmERV) present in mouse (*Mus musculus*) chromosome (chr) 7 [GenBank: AC167978]. To enhance one-step RT-PCR reactions, 2.5 μl of 1 μg/μl carrier RNA from QIAamp UltraSens™ Virus Kit (Cat. no. 53704) (QIAGEN) was added to the reaction mixtures of the one-step RT-PCR reactions as indicated in Figure 1. To examine whether the contaminant was RNA, 2 μl of 10 μg/ml RNaseA (Cat. no. 19101) (QIAGEN) were added in the one-step RT-PCR reaction mixture as indicated in Figure 1C. The RT-PCR was conducted in 25 μl (Kit I, Kit T, and Kit Q) or 25.5 μl (Kit P) of reaction mixture according to manufacturers' instructions.

By adding carrier RNA in the samples to enhance the RT-PCR reaction, we consistently detected a positive band using Kit I in negative controls using two primer sets (419F and 1154R, and GAG-I-F and GAG-I-R) which are widely used to amplify XMRV (Figure 1A). These results were confirmed by two independent laboratories (JRC and IVR) under the same experimental conditions. The positive reaction was observed in all four batches (derived from four different lots) of the kit tested. To exclude the possibility that water, the carrier RNA or the primers used were contaminated with an XMRV-like genome, we tested additional one-step RT-PCR kits, termed Kit P and Kit T, from two different manufacturers. Consequently, we could not detect positive bands utilizing these kits (Figure 1B) strongly suggesting that the component(s) of Kit I contained XMRV-like viral genomes. Most of the contaminants appeared to be RNA because the positive bands disappeared after adding RNaseA in the reaction mixture from Kit I (Figure 1C).

**Figure 2:**
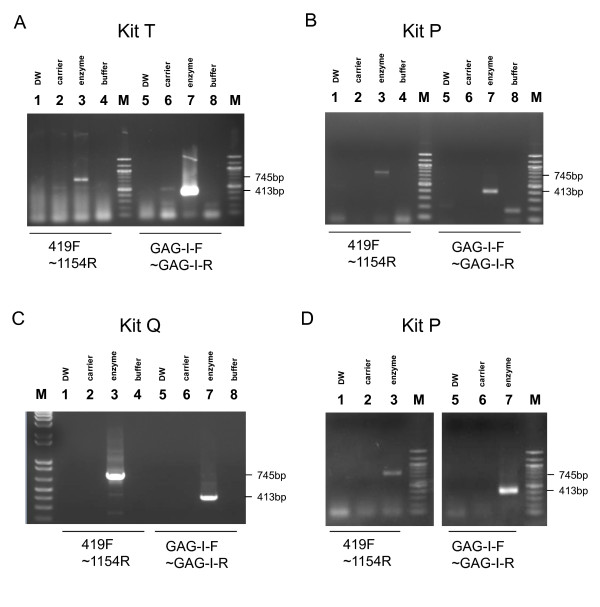
**One-step RT-PCR for identification of contaminants in Kit I and Platinum Taq**. (A-C) One-step RT-PCR for identification of a contaminated component in Kit I. The experiments were conducted in two independent laboratories, IVR and JRC. In IVR, nucleic acids were extracted from 50 μl of the enzyme mix of the RT-PCR Kit I using an RNA purification column (QIAamp viral RNA mini kit [Cat. no. 52904] [QIAGEN]) and the presence of polytropic endogenous MLV was examined by using the RT-PCR Kit T (A) and Kit P (B). In JRC, nucleic acids were extracted from 75 μl of the enzyme mix of RT-PCR Kit I using an RNA/DNA purification column (PureLink™ Viral RNA/DNA Kit [Cat. no. 12280-050] [Invitrogen]), and the presence of polytropic endogenous MLV was examined using Kit Q (C). Five μl of test samples were examined with primers indicated below the corresponding lanes. The RT-PCR conditions for Kit T and Kit P were the same as in Figure 1B. The RT-PCR conditions for Kit Q were as follows: reverse transcription at 50°C for 30 minutes; activation at 95°C for 15 minutes; 45 cycles of the following steps: 94°C for 30 s, 57°C for 30 s, and 72°C for 1 minute; and a final extension at 72°C for 10 minutes. Lanes 1 and 5, DW; lanes 2 and 6, column-purified carrier RNA (carrier); lanes 3 and 7, column-purified nucleic acids from enzyme mix (enzyme) of the Kit I; lanes 4 and 8, 1 μl buffer of the Kit I plus 4 μl DW (buffer). (D) One-step RT-PCR for the detection of MLV RNA in Platinum Taq. Nucleic acids were extracted from 50 μl of the Platinum Taq using an RNA purification column (QIAamp viral RNA mini kit [QIAGEN]) and the presence of MLV RNA was examined by using the RT-PCR Kit P. Five μl of test samples were examined with primers indicated below the corresponding lanes. The RT-PCR condition was the same as in Figure 1B with the exception of the PCR cycles (60 cycles instead of 45 cycles). Abbreviation; DW: distilled water. M: DNA size marker.

To further investigate the contaminant in Kit I, nucleic acids purified from the enzymes (a mixture of reverse transcriptase and *Taq *DNA polymerase) and the buffer contained in the kit were tested by adding the individual components to three different one-step RT-PCR kits (Kit T, Kit P, and Kit Q) (Figure 2A-C). As a result, we detected positive bands when the nucleic acids purified from the enzymes of Kit I were added to RT-PCR Kit T, Kit P or Kit Q using two primer sets (419F and 1154R, and GAG-I-F and GAG-I-R). On the contrary, we could not detect the presence of MLV genomes in the buffer of Kit I. These data indicated that the enzyme mixture of Kit I was contaminated with XMRV-like viral RNA.

**Figure 3:**
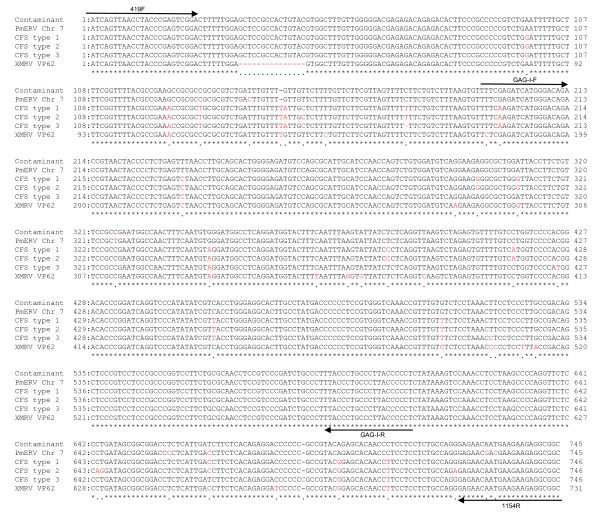
**Sequence alignments of a partial *gag *region of the contaminant in Kit I with a PmERV chr 7, XMRV strain VP62, and MLV-like sequences derived from CFS patients (CFS types 1 to 3)**. Origins of the sequences used for the alignment are described in the Findings. Sequence alignments were performed using GENETYX Win ver. 6 (GENETYX, Shibuya, Tokyo, Japan).

PCR products amplified using primers 419F and 1154R were cloned into pCR4Blunt -TOPO (Invitrogen) and sequenced for both strands. Three clones (two clones at JRC and one clone at IVR) were sequenced and found to be nearly identical (one nucleotide difference between one another). These sequences have a 9 nucleotide deletion observed in some endogenous polytropic MLVs in place of the XMRV-specific 24 nucleotide deletion in the 5' *gag *leader region and are nearly identical to polytropic endogenous MLVs encoded in multiple chromosomal locations of the C57BL/6J mouse genome. The nucleotide sequences of the representative clone [GenBank: AB597300] were aligned with sequences deposited in GenBank as follows: MLV-like virus from CFS patients types 1, 2 and 3 [GenBank: HM630562, HM630558, and HM630559] [13], XMRV strain VP62 [GenBank: NC_007815] [2] and one representative PmERV on chr 7 [GenBank: AC167978; nt 65,391-64,647] (Figure 3). The contaminant was nearly identical (99.4% identity) to the PmERV chr 7 with only 4 nucleotide differences in the sequenced region. In addition, the contaminant was quite similar (96.9-97.6% identity) to the MLV-like viral sequences (CFS types 1, 2, and 3) derived from CFS patients.

**Figure 4:**
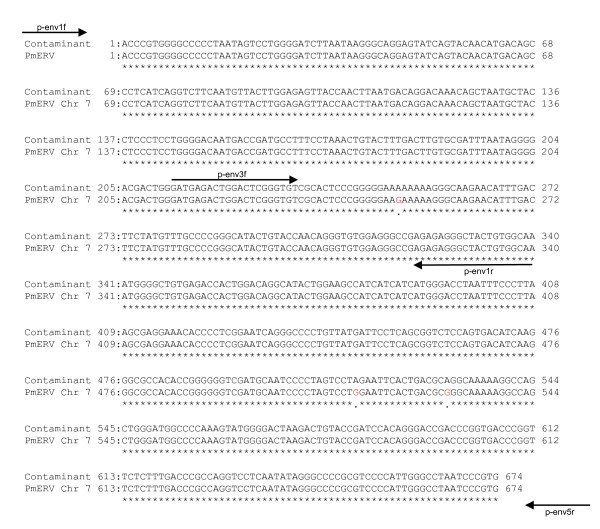
**Sequence alignments of a partial *env *region of the contaminant in Kit I with a PmERV chr 7**. Origins of the sequences used for the alignment are described in the Findings. Sequence alignments were performed using GENETYX Win ver. 6 (GENETYX, Shibuya, Tokyo, Japan).

To further characterize the contaminant, we conducted additional RT-PCRs (Figure 1D) amplifying partial *env *regions with two primer sets (p-env1f and p-env1r, and p-env3f and p-env5r) based on the sequence of the PmERV chr 7, and then sequenced the amplicons directly. We determined 674 bp of the N-terminal *env *region [GenBank: AB597301] and found that the contaminant was nearly identical (99.6% identity) to the PmERV chr 7 [GenBank: AC167978; nt 59,992-59,319] (Figure 4).

It should be noted that Kit I contains an anti-DNA polymerase monoclonal antibody to accomplish hot start-PCR and to reduce non-specific amplification. Mice have enormous copy numbers of endogenous retroviruses in their genomes; and hybridomas, for manufacturing monoclonal antibodies, have been found to produce high amounts of retroviral particles [16]. Therefore, we suspect that the *Taq *DNA polymerase in Kit I was contaminated with the endogenous MLVs. This possibility has been also pointed out by others [17,18]. Because the reverse transcriptase (SuperScriptIII) and the *Taq *DNA polymerase (Platinum Taq) in Kit I can be purchased separately from the manufacturer, we attempted to detect the MLV genome in the Platinum Taq polymerase using the same protocol as the one performed in Figure 2A-B. As a result, we detected MLV genomes in the Platinum Taq DNA polymerase using the RT-PCR Kit P and Kit T (Figure 2D for Kit P and data not shown for Kit T).

Surveys have been conducted by several research groups on XMRV infection in CFS patients, but the results have been inconsistent. Although all research groups carefully performed their experiments to test XMRV infection by PCR and/or RT-PCR, it is still difficult to conclude that the positive results linking XMRV with CFS are not laboratory artifacts. Xenotropic (or polytropic) MLVs are widespread, and there may be many opportunities for samples to get contaminated with such ubiquitous viruses in laboratories when conducting biological or medical research [17]. In this study, we evaluated several one-step RT-PCR kits and a *Taq *DNA polymerase for the contamination of MLV-related genomes and found that the test kit and the *Taq *DNA polymerase from Invitrogen were contaminated with MLV-related genomes.

The findings in the present study indicate that contaminating nucleic acids in the test kits can potentially produce false-positive PCR results in studies of XMRV and other MLV-related viruses. In particular, our results raise the possibility that the PCR products described by Lo et al. [13] were derived from contaminating MLV RNA and/or DNA. It should be noted, however, that in contrast to our data which shows MLV contamination even in water controls, their report demonstrated that polytropic MLV sequences were found more frequently in CFS patients than in healthy controls and not at all in water controls. Nonetheless, Lo et al. mentioned in the report that Platinum Taq from Invitrogen gave them the best results among *Taq *polymerases from several suppliers and was used to test the patient samples [13]. They also used Invitrogen's Superscript II RT and Platinum Taq for RT-PCR, albeit in a two-step cDNA synthesis and PCR amplification procedure [13]. As pointed out by Erlwein et al. in the comments [19] responding to the report by Lo et al. [13], assurance that control samples were assayed simultaneously with the positively identified ones in a blinded, randomized way was missing in their study, unfortunately.

The requirement for quality control to avoid contamination of endogenous retroviral genomes in test kits may differ depending on the intended purpose. However, in XMRV studies, many researchers conduct ultra-sensitive PCR or RT-PCR to detect extremely small amounts of XMRV. Therefore, in the investigation of XMRV infection, researchers should prudently evaluate test kits for the presence of genomes of endogenous MLV as well as XMRV.

## Competing interests

The authors declare that they have no competing interests.

## Authors' contributions

RAF, ES, and TM designed the experiments. RAF and ES performed the experiments. TM wrote the manuscript. All authors read and approved the final manuscript.

## References

[B1] Urisman A Molinaro RJ Fischer N Plummer SJ Casey G Klein EA Malathi K Magi-Galluzzi C Tubbs RR Ganem D Silverman RH DeRisi JL Identification of a novel Gammaretrovirus in prostate tumors of patients homozygous for R462Q RNASEL variant PLoS Pathog 2006 2 e25 10.1371/journal.ppat.0020025 16609730 PMC1434790

[B2] Dong B Kim S Hong S Das Gupta J Malathi K Klein EA Ganem D Derisi JL Chow SA Silverman RH An infectious retrovirus susceptible to an IFN antiviral pathway from human prostate tumors Proc Natl Acad Sci USA 2007 104 1655 1660 10.1073/pnas.0610291104 17234809 PMC1776164

[B3] Lombardi VC Ruscetti FW Das Gupta J Pfost MA Hagen KS Peterson DL Ruscetti SK Bagni RK Petrow-Sadowski C Gold B Dean M Silverman RH Mikovits JA Detection of an infectious retrovirus, XMRV, in blood cells of patients with chronic fatigue syndrome Science 2009 326 585 589 10.1126/science.1179052 19815723

[B4] Erlwein O Kaye S McClure MO Weber J Wills G Collier D Wessely S Cleare A Failure to detect the novel retrovirus XMRV in chronic fatigue syndrome PLoS One 2010 5 e8519 10.1371/journal.pone.0008519 20066031 PMC2795199

[B5] Groom HC Boucherit VC Makinson K Randal E Baptista S Hagan S Gow JW Mattes FM Breuer J Kerr JR Stoye JP Bishop KN Absence of xenotropic murine leukaemia virus-related virus in UK patients with chronic fatigue syndrome Retrovirology 2010 7 10 10.1186/1742-4690-7-10 20156349 PMC2839973

[B6] Hong P Li J Li Y Failure to detect Xenotropic murine leukaemia virus-related virus in Chinese patients with chronic fatigue syndrome Virol J 2010 7 224 10.1186/1743-422X-7-224 20836869 PMC2945957

[B7] McClure M Wessely S Chronic fatigue syndrome and human retrovirus XMRV BMJ 2010 340 c1099 10.1136/bmj.c1099 20185494

[B8] Switzer WM Jia H Hohn O Zheng H Tang S Shankar A Bannert N Simmons G Hendry RM Falkenberg VR Reeves WC Heneine W Absence of evidence of xenotropic murine leukemia virus-related virus infection in persons with chronic fatigue syndrome and healthy controls in the United States Retrovirology 2010 7 57 10.1186/1742-4690-7-57 20594299 PMC2908559

[B9] van Kuppeveld FJ de Jong AS Lanke KH Verhaegh GW Melchers WJ Swanink CM Bleijenberg G Netea MG Galama JM van der Meer JW Prevalence of xenotropic murine leukaemia virus-related virus in patients with chronic fatigue syndrome in the Netherlands: retrospective analysis of samples from an established cohort BMJ 2010 340 c1018 10.1136/bmj.c1018 20185493 PMC2829122

[B10] Dolgin E Chronic controversy continues over mysterious XMRV virus Nat Med 2010 16 832 10.1038/nm0810-832a 20689529

[B11] Enserink M Chronic fatigue syndrome. Conflicting papers on hold as XMRV frenzy reaches new heights Science 2010 329 18 19 10.1126/science.329.5987.18 20595585

[B12] Enserink M Chronic fatigue syndrome. New XMRV paper looks good, skeptics admit--yet doubts linger Science 2010 329 1000 10.1126/science.329.5995.1000 20798285

[B13] Lo SC Pripuzova N Li B Komaroff AL Hung GC Wang R Alter HJ Detection of MLV-related virus gene sequences in blood of patients with chronic fatigue syndrome and healthy blood donors Proc Natl Acad Sci USA 2010 107 15874 15879 10.1073/pnas.1006901107 20798047 PMC2936598

[B14] Barnes E Flanagan P Brown A Robinson N Brown H McClure M Oxenius A Collier J Weber J Günthard HF Hirschel B Fidler S Phillips R Frater J Failure to detect xenotropic murine leukemia virus-related virus in blood of individuals at high risk of blood-borne viral infections J Infect Dis 2010 202 1482 1485 10.1086/657167 20936982

[B15] Cornelissen M Zorgdrager F Blom P Jurriaans S Repping S van Leeuwen E Bakker M Berkhout B van der Kuyl AC Lack of detection of XMRV in seminal plasma from HIV-1 infected men in The Netherlands PLoS One 2010 5 e12040 10.1371/journal.pone.0012040 20706581 PMC2919391

[B16] Shepherd AJ Wilson NJ Smith KT Characterisation of endogenous retrovirus in rodent cell lines used for production of biologicals Biologicals 2003 31 251 260 10.1016/S1045-1056(03)00065-4 14624795

[B17] Weiss RA A cautionary tale of virus and disease BMC Biol 2010 8 124 10.1186/1741-7007-8-124 20920148 PMC2946284

[B18] Silverman RH Nguyen C Weight CJ Klein EA The human retrovirus XMRV in prostate cancer and chronic fatigue syndrome Nat Rev Urol 2010 7 392 402 10.1038/nrurol.2010.77 20517289

[B19] Kaye S Robinson M McClure M Chronic fatigue syndrome: xenotropic murine leukemia virus-related virus, murine leukemia virus, both, or neither? Proc Natl Acad Sci USA 2010 107 E161 10.1073/pnas.1012441107 20884850 PMC2972973

